# The Furr-Recovery Method: Interacting with Furry Co-Workers during Work Time Is a Micro-Break That Recovers Workers’ Regulatory Resources and Contributes to Their Performance

**DOI:** 10.3390/ijerph192013701

**Published:** 2022-10-21

**Authors:** Ana Junça-Silva

**Affiliations:** 1 Business Research Unit (BRU-IUL), ISCTE—Instituto Universitário de Lisboa, 1649-026 Lisboa, Portugal; ana_luisa_silva@iscte-iul.pt; 2 Superior School of Management, IPT—Instituto Politécnico de Tomar, 2300-313 Tomar, Portugal

**Keywords:** recovery, micro-breaks, performance, pets, human-animal interactions

## Abstract

Drawing on the conservation of resources theory and the recovery step model our research expands on a cognitive (regulatory resources) mechanism that links human–animal interactions and employee performance. This study aimed to explore whether daily human–animal interactions during worktime would be conceived as a daily-recovery process that restores the individual’s daily regulatory resources and, as a result, improves daily adaptive and task performance. To test this, a daily diary study during 10 working days, with 105 teleworkers was performed (*N* = 105 × 10 = 1050). Multilevel results demonstrated that daily interactions between human and their pets served to recover their daily regulatory resources that, in turn, improved daily task-and-adaptive performance. This research not only expands our theoretical understanding of regulatory resources as a cognitive mechanism that links human-animal interactions to employee effectiveness but also offers practical implications by highlighting the recovery role of interacting with pets during the working day, as a way to restore resources needed to be more effective at work.

## 1. Introduction

Pets trigger solace, even if they are “speechless friends” … “The word can’t go where the heart can, not completely. It’s freeing, to think there’s always an aspect of us outside the grasp of speech, the common stuff of language” [1] (p. 48).

The COVID-19 crisis gave rise to an increasing number of organizations adopting telework as a strategy to contain the virus widespread and assure their productivity [2]. Telework is a flexible work arrangement in which workers may work from other locations (e.g., home) through the support of information and communication technologies [2,3].

With the social distance imposed by the COVID-19 outbreak, pet owners took the opportunity of teleworking nearby their furry friends, making them their “furry co-workers”. By having the opportunity to work nearby their side, interacting with furry co-workers become more frequent (e.g., head petting or taking the pet for a walk), as they had the chance to take micro-breaks to do it [4]. According to the recovery step model [5], micro-breaks are frequent and needed during the working day as they may restore the energy needed to perform the tasks efficiently. Micro-breaks are short and informal breaks/respite activities taken voluntarily between tasks [6] (p. 773), are generally considered more flexible in timing, duration, and frequency and are typically self-initiated [5]. Micro-breaks may include coffee breaks or tea breaks. By engaging in these kinds of micro-breaks, employees can recover their energy and regulatory resources—the capacity to exert self-control over diverse behaviors, emotions, and impulses (e.g., allocate and redirect cognitive attention to the tasks even when tired or fatigued) [7]; and subsequently function at full capacity [8] which leads to increased performance [9]. Despite the diverse empirical demonstrations of micro-breaks during worktime for performance-related outcomes [10], no study has explored human–animal interactions (HAIs) during work as a micro-break.

Human–animal interactions have been recently acknowledged as a hot topic for organizational behavior as pets may help their humans to be, feel and perform better [4]. As Kelemen et al. [11] noted pets intersect organizational life in many ways. HAIs describe a wide spectrum of interactions and relationships between animals and humans [12] and may include physical (e.g., head pet the furry friend), affective (e.g., observing the furry friend playing with a toy), or cognitive (e.g., perceived support by the furry friend). HAIs have been demonstrated to improve health [13] and well-being [14] and reduce stress and anxiety [15]. However, the role that HAIs may play at work has been less investigated; indeed, there is a consensus that further research investigating the links between HAIs at work is needed [4,16].

Based on the recovery step model, we argued that HAIs during work time are micro-breaks that may restore the individuals’ regulatory resources by making them experience pleasure (i.e., contributing to relaxing) and distracting them from work (i.e., detaching them from work, at least, by moments). Moreover, according to the conservation of resources perspective [17], we expected that by recovering regulatory resources, individuals are able to focus and concentrate on their tasks, thereby contributing to their improved task performance. We also defended that when regulatory resources are replenished, individuals feel full of resources which makes them more adaptable to uncertain and challenging demands, thus contributing to their adaptive performance.

This study contributes to expanding the literature on HAIs at work and their work-related outcomes. First, examining how employees’ interactions with their pets influence their performance will help scholars and managers to better understand how teleworkers may have access to unique resources—that they would not be able to access if they were working at the office—in the home domain. Second, telework is increasingly being adopted by many organizations across the world; thus, managers need to understand how resourceful it may be for employees who own pets. Third, demonstrating that interacting with furry co-workers while teleworking may be conceived as a micro-break is of relevant importance for the literature on recovery at work, once so far these interactions have been disregarded as micro-breaks. These micro-breaks may provide the support necessary for workers to recover and replenish their regulatory resources that, in turn, may serve as a cognitive mechanism linking HAIs to employee effectiveness.

## 2. Theoretical Framework

### 2.1. The Importance of Human–Animal Interactions

Pets are ubiquitous to human life and permeate a diversity of social and familiar contexts improving healing, and offering support, attachment, confidence, caring, and companionship [18]. Indeed, they are occupying crucial roles for their humans in many interpersonal and intra-familial relationships [19], as they have been demonstrated to represent an individual’s support system and facilitators of well-being, happiness, and a sense of safety [20]. This may explain the increasing number of pets in household families. For instance, The American Veterinary Medical Association [21] estimated that 57% of U.S. households owned a pet, including 76.8 million dogs, 58.3 million cats, birds, horses, and other companion animals. In addition, the social representation of pets has also changed. For example, Junça-Silva [14] demonstrated that dog and cat owners viewed their pets as family members, best friends, or their “furry babies”. Hence, pets are conquering a time and a special space in the heart of modern families. These shreds of evidence justify the need to understand deeper how HAIs may trigger benefits for individuals.

Human–animal interactions (HAIs) are not a new topic; however, in the field of organizational behavior or organizational psychology, it is considered a hot topic [4,11]. HAIs have been often defined as all the interactions between humans and non-human beings [22] and may include physical (e.g., head petting the furry friend), affective (e.g., observing the furry friend playing with a toy), or cognitive interactions (e.g., perceived support by having the furry friend nearby) [4,23].

There is a great amount of research demonstrating the benefits of HAIs for an individual’s daily life [14]. For instance, diverse studies have consistently shown that HAIs increase the quality of social interactions, and reduce perceived loneliness, and psychological symptoms such as depression and stress, e.g., [23,24,25]. In addition, HAIs also appear to decrease negative affect after stressful situations, e.g., [26]. Moreover, HAIs have also been demonstrated as slowing the development and progression of chronic illnesses by decreasing loneliness, anxiety, and sympathetic nervous system arousal, and improving physical fitness by increasing motivation to exercise [27].

While most of these studies were conducted under medical or veterinary disciplines, fewer studies have devoted their attention to understanding if HAIs may have the same impact on work-related contexts.

### 2.2. HAIs in the Work Context

Recently, some scholars have recognized that pets intersect with daily life at work in many ways e.g. [11,28,29]. Moreover, practitioners have also recognized that pet-friendly practices improve the levels of work engagement, happiness, and performance [11], given the increasing number of organizations implementing it in their organizational strategies [28].

One of the most common pet-friendly practices is telework, in part because many organizations are not physically prepared to receive their workers’ “furry babies” [14,30], and also because the COVID-19 pandemic situation imposed telework as a strategy to contain the virus widespread [1]. Nilles [31] proposed the terms telecommuting and teleworking to contextualize telework. The difference is that teleworking is more comprehensive than telecommuting, since teleworking means any form of work, through information technologies, other than in the workplace, which can be from any point (e.g., home, or another branch of the company) [31]. On the other hand, telecommuting just means working from home, without any kind of displacement [32].

After the COVID-19 outbreaks, some organizations continued to use telework as it seems to improve their workers’ well-being and task (completing the formally assigned tasks) and adaptive performance (being able to adapt to changes in routines, procedures, or resources to accomplish the job) [33]. Considering this, some studies demonstrated that a great percentage of pet owners preferred to telework than work at the office [34]. Telework has been increasingly valued by individuals who own pets because working from home allows them (1) to spend more time with their “furry babies”, (2) avoid leaving them alone for many hours, and (3) minimize their concerns with their “furry babies” during the working day which also allows for a better concentration on the tasks at hand [4], and makes them happier [14].

The conservation of resources theory (COR) [17] may support this evidence. A central tenet of the COR is that individuals strive to protect, acquire, and maintain their personal resources. Once resources are lost, individuals engage in strategies to reestablish them [35], for instance taking an enjoyable micro-break from work. When their strategies are succeeding, then they become full of resources that, in turn, enable their ability and concentration on what they have to accomplish. On the opposite, when they fail in restoring their resources, they tend to be stressed about it which impairs their focus on the tasks at hand [35].

Despite the fewer studies on HAIs in the work context, we argue that these interactions may provide the individual with the resources lost in their daily work demands. First, interacting with pets has been associated with increased well-being [14], positive emotions [4], and emotional support [23,24]. It has also been shown to decrease the perception of loneliness [27], stress [22], and anxiety [22]. Positive emotional states are resourceful as they help individuals to expand their set of thoughts and actions [17]. By experiencing positive emotional states when interacting with their pets (e.g., satisfaction, calmness), individuals are likely to recover resources needed for effective performance. Thus, interacting with their “furry co-worker” may help individuals to replenish their resources which may improve their ability to focus on what they have to do. Based on the COR theory we hypothesized the following.

**Hypothesis** **1:** *There will be a positive relationship between daily HAIs and daily regulatory resources.*

### 2.3. The Indirect Effect of Daily Regulatory Resources

As we mentioned before, HAIs appear to have several affective, attitudinal, and behavioral benefits for humans e.g., [27]. However, as far as we know, no study has explored the effects of HAIs as a source of resources. We argue that HAIs may be a source of cognitive resources for the individual, that help him/her to restore other ones, such as energy—a resource linked to self-regulatory resources—the regulatory capacity to exert self-control over a variety of behaviors, emotions, and impulses [36]. These cognitive resources are crucial for individuals’ working days as they need them to perform their tasks that require effortful regulation of their affect and cognition [37,38]. Moreover, these resources are limited because individuals during their work spend some of them on different self-control activities (e.g., suppressing and displaying emotions; allocating and redirecting cognitive attention to the tasks even when are tired) [39,40]. When this happens, individuals must stop regulatory acts and rest to recover the depleted resources before tackling the next set of self-regulation tasks [37].

The recovery step model argues that micro-breaks are crucial for individuals to rest and recover lost resources, such as regulatory ones [5], through recovery experiences (i.e., control, relatedness, mastery, enjoyment, detachment, and relaxation) [37]. Indeed, micro-breaks are a way to cease continual resource consumption and renew energy [5], serving as a resource replenishing strategy as they refer to short respites that are taken informally and voluntarily when needed between task episodes [6,37]. Empirically there is some support for this as some studies have consistently demonstrated that taking discretionary breaks throughout the working day decreases fatigue and improves resources needed for efficient performance, see [41,42]. Overall, it seems that even short respite activities during breaks can be effective energy management strategies to reenergize employees while at work [5].

Among the diverse kinds of micro-breaks explored in the literature (e.g., coffee breaks, tea breaks, lunch breaks) no study has considered HAIs as a micro-break. We argue that HAIs may help individuals to regain self-regulatory resources and in turn, this increase in resources may benefit workers’ performance. First, research has shown that with eye contact between humans and pets, individuals tend to feel supported, and connected, and experience positive emotions [43]. Positive emotions are resources that broaden an individual’s cognitive and action repertoire leading to the acquisition of enduring resources [44]. Second, touching pets is both physiologically and emotionally pleasurable. Olmert [45] asserted that the urge to touch an animal is biological. For instance, neuroscience research has demonstrated that looking at a dog, stroking or talking to him/her, can arouse oxytocin—a hormone that triggers feelings of pleasure and relieves stress or other negative affective states. Indeed, oxytocin bolsters one’s immune system, decreases the production of stress hormones, and consequently lessens feelings of fear and danger [45]. By making individuals feel good, oxytocin may also help them to broaden and restore other resources such as energy and cognitive resources needed to self-regulate work behavior. Third, pets may be a source of personal resources due to their role in supporting, caring, and creating bonds with their humans. Indeed, from an attachment standpoint, attachment experiences are processed and stored in the right hemisphere of the brain, influencing affective and cognitive functioning such as self-regulation [46]. For instance, familiars from the recently passed away, Queen Elisabeth II, have reported that in moments of anguish and stress the Queen resorted to her furry friends as she saw in them the perfect friend—even furry—to support and relieve her pain. Her family named it the dog mechanism “[…] If the situation becomes too difficult, she will sometimes literally walk away from it and take the dogs out” [47].

In sum, based on the recovery step model, we argue that HAIs may be a micro-break that restores the individuals’ regulatory resources helping them to deal effectively with their daily work demands, and as such perform better. Based on the arguments above, interacting with pets appears to be pleasurable and beneficial to individuals; therefore, it may allow them to relax and thereby recover regulatory resources needed to effectively perform. In addition, interacting with pets can distract employees from work-related thoughts and concerns, thereby enhancing detachment. Relaxation and detachment represent unwinding processes and therefore they put no further demands on resources and allow for regulatory resources to recover. As stated earlier, self-regulatory resources are crucial for performance [38], as they may improve the ability to focus, and the energy needed to adapt. To complete job tasks, or to adapt to changes in daily routine due to work-related hassles employees need their self-regulatory resources [38,40]. Hence, when they are with a low level of regulatory resources, employees may not have the self-control needed to sustain work and adapt to new challenges.

**Figure 1:**
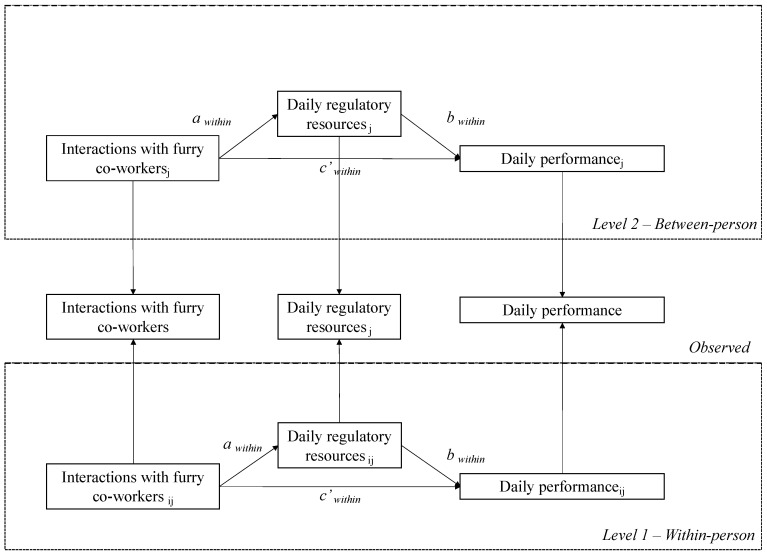
Conceptual model. For brevity, the control variables were not shown in the framework.

The following hypothesis was thereby defined (see Figure 1).

**Hypothesis** **2:** *The relation between daily HAIs and daily (a) task and (b) adaptive performance will be mediated by daily regulatory resources.*

## 3. Method

### 3.1. Participants and Procedure

One hundred and five Portuguese teleworkers took part in the study. Most teleworkers were researchers (43%), human resources managers (18%), managers (15%), designers (12% and advertisers (12%). Overall, 58% were female, the mean age was 33.70 years old (SD = 12.71), and the mean tenure was 14 years (SD = 4.56). On average, they worked about 35 h per week (SD = 14). All participants had pets (M = 3; SD = 4.10) living with them. Mostly they reported having dogs (87%) followed by cats (33%). On average, participants reported having pets at 12 years (SD = 10.41).

Teleworkers from the researcher’s professional network were asked by email to participate in a study about telework attitudes. The ones that agreed to participate received a second email in which the main goals of the study and the data collection procedure were clarified. They were also assured that their participation was completely voluntary and anonymous and that their responses were confidential. Then, they signed an informed consent form before answering the general survey. After this, they received the hyperlink for the general survey. This aimed to assess demographic characteristics and pets’ characteristics. In the next week, they started the daily questionnaires (collected once per day at the end of the working day) for 10 days (from Monday to Friday for two weeks). Each participant received a daily email, at the end of the day, at 6 pm with the hyperlink for the survey. They had to answer it by 11 pm. Of the 150 teleworkers that agreed to participate, 105 provided valid responses across the 10 days (*n* = 1050; response rate: 70%).

### 3.2. Measures

Human–animal interactions were measured with four items developed by Junça-Silva et al. [4]. The items were “Today while teleworking I took breaks to interact with my pet”; “While I worked from home today, my pet was close to me”; “Today, while teleworking, I stopped working to pet my furry friend”; “While I was working today, I interacted with my pet”). Participants used a 5-point scale (1 never; 5 four times or more). Multilevel reliability through the Alpha and the Omega index were good (α_between_ = 0.80, ω_between_ = 0.84; α_within_ = 0.90, ω_within_ = 0.89).

Daily regulatory resources. To assess regulatory resources, the 3-item Regulatory Resource Availability scale [48] was used (e.g., “Today, I have not been feeling mentally energetic.”). Responses were given on a 5-point Likert scale ranging from: (1) Never to (5) Always. Multilevel reliability tests indicated acceptable reliability (α_between_ = 0.85, ω_between_ = 0.86; α_within_ = 0.80, ω_within_ = 0.78).

Daily performance. We used 10 items from the individual work performance questionnaire [49] to assess the task (five items: e.g., “Today, I managed to plan my work so that it was done on time”) and adaptive performance (five items; “Today, “I have demonstrated flexibility”). Responses were given on a five-point Likert scale ranging from: (1) Seldom to (5) Always. (α_between_ = 0.78; 0.88, ω_between_ = 0.75; 0.82; α_within_ = 0.86, 0.87, ω_within_ = 0.86, 0.88).

Control variables. The time of data collection (from Monday to Friday) was a daily-level control variable once it was found that it influences performance and regulatory resources [4]. Sex and number of pets were person-level control variables because the number of pets may influence daily HAIs and subsequent regulatory resources (as it may lead to a higher number of volatile actions to interact with them), and sex may influence both regulatory resources and performance-related outcomes.

### 3.3. Data Analysis

This study used multi-level analysis with nested data to examine the underlying model. First, we calculated the analysis of variance components. Between-person variance represents the relative differences among participants’ overall variable levels whereas within-person differences represent a participant’s change in a particular variable from one day to the next. We found that there was significant variance in daily HAI (ICC = 0.80), regulatory resources (ICC = 0.85), task (ICC = 0.87), and adaptive performance (ICC = 0.85). This evidenced that these variables have significant variation both at within and between-person levels. Thus, we proceeded with the multilevel analysis.

As both the predictor, the mediator, and the criterion variables were measured at the same time, we took some measures to avoid the issue of common method variance. First, we shuffled the questions of various measures and then used various dummy questions (e.g., I like ice creams). Second, we tested the factorial structure of the data through multilevel CFAs using R. 4.2.1 (Auckland University, Auckland, New Zeland). We first tested a four-factor model with the four multi-item variables under study (HAIs, self-regulatory resources, task, and adaptive performance). The four-factor model proposed yielded a good fit (χ^2^ = 408.91; *p* < 0.001; df = 96; RMSEA = 0.09; CFI = 0.99; SRMR_within_ = 0.06; SRMR_between_ = 0.06). The model fitted better than a three-factor model (where task and adaptive performance were loaded on one factor; χ^2^ = 1110.45; *p* < 0.001; df = 96; RMSEA = 0.16; CFI = 0.97; SRMR_within_ = 0.12; SRMR_between_ = 0.13), a two-factor model (where task and adaptive performance were loaded on one factor and HAIs and self-regulatory resources loaded on the other; χ^2^ = 2702.84; *p* < 0.001; df = 79; RMSEA = 0.27 CFI = 0.95; SRMR_within_ = 0.23; SRMR_between_ = 0.24), and a one-factor model (where all items were loaded on one factor; χ^2^ = 5611.34; *p* < 0.001; df = 80; RMSEA = 0.38; CFI = 0.91; SRMR_within_ = 0.36; SRMR_between_ = 0.37). Thus, the current four-factor structure was valid. These results together with the Cronbach alpha and Omega reliability scores across all the measurement scales evidenced the discriminant and convergent validity of the study; hence, we proceeded with the test of hypotheses.

The hypothesis was tested through the macro–multilevel mediation (MLMed), in SPSS version 28 (IBM Corp., Armonk, NY, USA) [50]. This macro appears to deliver similar results, in the estimation of model’ parameters, to what other software alternatives do (e.g., Mplus). MLMed tests both fixed and random effects. A fixed effect for the intercept specifies one intercept for all participants in the model, and a random effect allows participants’ intercepts to vary. Fixed effects of slopes are simple relations between two variables that apply across people (e.g., daily HAIs are positively related to task performance for everyone in the same way). Random effects of slopes, however, indicate whether there is significant variance in the slope of a given fixed effect from person to person. If there is a significant random effect, daily HAIs do not relate to task performance in the same way for each person. We tested fixed effects for each predictor variable and random effects for each within-person predictor variable. We also allowed a random effect for the intercept. We standardized all variables before entering them into MLmed so that we could compare relative effect sizes. MLmed provides output for the 1-1-1 mediation model, with daily HAIs predicting the daily regulatory resources, and an output for the outcome model, with daily regulatory resources predicting daily task, and adaptive performance. Therefore, we included these models in the results to examine how variables predicted each other across the model. The MLmed macro also calculates 95% Monte Carlo confidence intervals, based on 10,000 samples, for the indirect effect. These confidence intervals are significant when they do not include zero. Thus, the MLMed is a suitable macro to test our 1-1-1 multilevel mediation model.

## 4. Results

### 4.1. Descriptive Statistics

Table 1 shows the descriptive statistics and correlations.

### 4.2. Hypothesis Testing

As we mentioned before, to test our hypotheses, we considered the hierarchical structure of the data, in which daily data were nested within individuals. We centered our variables before the analysis.

Hypothesis 1 expected that daily HAIS would positively influence daily self-regulatory resources. Daily HAIS positively correlated with daily self-regulatory resources (γ = 0.09, *p* < 0.001), lending support to the first hypothesis.

Hypothesis 2 expected that daily HAIS would positively influence daily task-and-adaptive performance through daily regulatory resources. The results showed a significant indirect effect of daily HAIs on daily task performance via daily regulatory resources, at the within-person (Estimate_within_ = 0.03, *p* < 0.05, 95% CI [0.01, 0.04]) and between-person level (Estimate_between_ = −0.08, *p* < 0.001 95% CI [−0.13, −0.03] (see Table 2).

Moreover, the results showed a significant indirect effect of daily HAIs on daily adaptive performance via daily regulatory resources, at the within-person level (Estimate_within_ = 0.02, *p* < 0.05, 95% CI [0.01, 0.03]), and between-person level (Estimate_between_ = −0.03, *p* < 0.05, 95% CI [−0.06, −0.01] (see Table 2). Thus, h1 was supported by the data.

## 5. Discussion

This study uses a daily diary method to extend the theoretical understanding of daily micro-breaks for employee performance, by integrating the conservation of resources theory [17], and the recovery step model [5] to develop a framework that conceives HAIs as a micro-break and explores its effect on employees’ task and adaptive performance via their regulatory resources. So far as we know, this is the first study that conceives HAIs during work time as a micro-break that may influence how workers perform their tasks and adapt to their daily challenges. Hence, this study expands the recovery step model by including HAIs as a micro-break. Moreover, by analyzing daily regulatory resources as a within-person mediator, this research highlights the role of a cognitive (regulatory resources) mechanism that links human-animal interactions and employee performance.

### 5.1. Theoretical Implications

First, the findings show a different pattern of results at the between and within-person levels. That is, while at the within-person level the indirect effect of HAIs on task and adaptive performance through regulatory resources is positive, at the between-person level, we find a negative effect. In other words, employees appear to have different needs to interact with their companion animals throughout the day, and at these times—when they really need—interacting with them seems to be beneficial as it leads to an increase in their regulatory resources, which, in turn, translates into (task and adaptive) performance improvements. That is, interacting with their “furry coworkers” appears to be beneficial for employees, and can be conceptualized as a pleasant micro-break capable of restoring the regulatory resources necessary for effective performance. Hence, interacting with “furry coworkers” appears to be pleasurable and beneficial to teleworkers; making a break to interact with their pet, appears to relax individuals and help them to recover the regulatory resources needed to effectively perform. In addition, “furry coworkers” can distract employees from work-related thoughts and concerns, thereby enhancing detachment. Relaxation and detachment are unwinding processes and therefore they allow for regulatory resources to recover which may energize and deliver the self-control necessary for individuals to complete their tasks and adapt to challenges and daily demands.

However, these micro-breaks should only occur when the individuals indeed need them—for example, when resources are running out in such a way that limits the concentration needed for the tasks at hand. This result can be related to the flow of tasks or the daily challenges and demands of the worker on that working day. That is, the more a working day is resource-draining, the more demands and challenges it presents. During these days, taking micro-moments to interact with pets seems to be resource-restorative, enough to improve the individual’s performance. These results are supported by several studies that have shown that the benefits of micro-breaks depend on the level of demands experienced by the individual throughout the day [5]. That is, micro-breaks may not be beneficial to recover energy when the individual is not experiencing a loss of resources; hence, in these days, taking successive breaks can be counterproductive and lead to the expenditure of regulatory resources.

The ego-depletion theory is in line with this finding as it posits that any kind of volitional act that requires self-control can deplete one’s resources [40]. Because individuals own a limited pool of inner resources [7] when engaging in successive self-regulatory actions (e.g., pet the furry baby), this available energy may be depleted [39]. The recovery process is an “unwinding and restoration process during which a person’s strain level that has increased as a reaction to a stressor or any other demand returns to its pre-stressor level” [41] (p. 366). Hence, by engaging in micro-breaks such as interacting with pets, individuals recover their energy lost when they experience a day full of demands [6], and subsequently function at full capacity [8]. However, when they do not lose resources, or at least do not lose a certain level of resources engaging in micro-breaks may lead to a decreased level of self-regulatory resources, and as a result an impairment in performance levels.

Overall, we can conclude that when teleworking, individuals who own pets may benefit from their presence, as these may be their “furry co-workers”, capable of not only giving love, caring, and affection [27], but also supporting the recovery of important resources, such as the regulatory ones, for their owners’ performance. This may be called the *furr-recovery method*—a method based on a micro-break aimed at recovering resources needed for employees’ daily effective performance. However, this *furr-recovery method* appears to be time and context-sensitive due to the different patterns of results found—the positive within-person and the negative between-person effect. Hence, the method is only beneficial when the individual is in truly need of recovery, otherwise, it may be a way to deplete self-regulatory resources that are crucial for effective performance.

### 5.2. Practical Implications

These results shed light on relevant findings for applied purposes. First, as a great amount of research has demonstrated, appears to be beneficial for workers’ happiness and performance [32,51]. This may be explained, at least, from a pet-owner perspective. For instance, some studies demonstrated that workers prefer to work from home, and would like to work from home more often, and this preference is intensified by pet owners [52]. Additionally, this study expands this justification by highlighting the role that HAIs may have as a beneficial micro-break especially when employees are experiencing a greater loss of resources. Hence, engaging in interacting with their furry co-workers (for instance, petting them) may replenish them with the energy and resources needed to perform. Thereby, managers may consider implementing at least a hybrid telework system as a strategy to improve their worker’s performance and well-being.

Moreover, if interacting with pets at home is beneficial, so will it be at the office. As such, managers may consider (when applicable) implementing pet-friendly practices such as creating a pet day at work, in which their workers are allowed to take their pets to work. Of course, not all organizations are physically prepared for this, and when this is not possible, telework may be a suitable strategy to motivate and engage their employees with their work.

### 5.3. Limitations and Future Directions

This study, despite its innovative nature, has some limitations to knowledge. The first one is related to the self-reported nature of the data which might lead to the common method bias [52]. However, as we collected data on individual experiences during teleworking there was no other way to measure the variables under study (e.g., HAIs). Hence, even with limitations, the self-reported data were the only way to access the individual’s personal experiences. The other limitation is related to the design of the study. Despite, using a daily diary method, we only collected data at a one-time point per day, which may limit the conclusions regarding the causality between the variables. Future studies should conduct similar studies but using different time points per day.

These results open new research venues for further investigations. First, it should be interesting to explore the contextual conditions under which HAIs may be beneficial. That is, what daily demands and daily hassles may trigger the individual’s need to interact with his/her “furry co-worker”. These would expand the furr-recovery method and its applicability. Second, the between-person-level relationships should be further explored because there may be individual differences accounting for such results. For instance, the big-5 dimensions or the dark personality may moderate the indirect path between daily HAIs and performance via regulatory resources. At last, an individual’s level of commitment and engagement may also explain the between-person-level results. For instance, employees who frequently interact with their pets at the between-person level may be those who are less involved or dedicated to their work and this study cannot control for this cross-level effect. Hence, future studies could look at possible cross-level effects.

## 6. Conclusions

This study gives insights into a new method to recover resources: the furr-recovery method—a method focused on interacting with “furry co-workers” during work time as a way to recover daily regulatory resources and improve employees’ performance. The furr-recovery method involves interacting with a “furry co-worker”. These furry interactions appear to be a pleasurable and relaxing micro-break that helps employees to recover their self-regulatory resources through these moments of detachment from work; in turn, individuals become energized and self-controlled enough to focus on their tasks and improve their adaptive performance even when challenges arise.

## Figures and Tables

**Table 1:** Means, standard deviations, and between-and within-person level correlations.

Variables	M	SD	1	2	3	4	5	6	7
1. HAIs	1.61	1.07	-	0.06 *	0.03	0.12 **	0.06 *	0.22 **	0.10 **
2. Regulatory resources	3.69	0.96	0.07 *	-	0.41 ***	0.24 **	0.03	0.01	−0.06 *
3. Task performance	3.85	0.86	0.04	0.13 **	-	0.42 ***	−0.06 *	0.03	0.06 *
4. Adaptive performance	3.70	0.83	0.02	0.21 ***	0.60 ***	-	−0.08 **	0.03	−0.02
5. Time	-	-	0.14 **	0.05	0.03	0.03	-	0.04	0.02
6. Number of pets	3.00	4.10	0.08	0.06	0.09 **	0.01	0.04	-	0.05
7. Sex	-	-	0.06	−0.02	0.02	−0.05 *	0.01	0.05	-

*Note*. Correlations below the diagonal are between-person levels. Correlations above the diagonal are within-person level. Sex: 1 = Male; 2 = Female. *N*_(observations)_ = 1050; *n*_(participants)_ = 105. *** *p* < 0.001, ** *p* < 0.01, * *p* < 0.05.

**Table 2:** Parameter estimates for 1-1-1 multilevel mediation model.

	Model 1 Mediator (Daily Regulatory Resources)	Model 1 Dependent (Daily Task Performance)	Model 2 Mediator (Daily Regulatory Resources)	Model 2 Dependent (Daily Adaptive Performance)
Within-level (L1) Effects				
Mean Intercept	2.37 ***	1.62 ***	2.37 ***	2.16 ***
Daily HAIs	0.09 **	−0.03	0.09 **	0.03
Regulatory resources	-	0.23 ***	-	0.16 ***
Time	0.01	−0.03 *	0.01	−0.05 ***
Number of pets	-	-	-	-
Sex	-	-	-	-
Between person Effects				
Daily HAIs	−0.21 ***	−0.05	−0.20 ***	0.10 *
Regulatory resources	-	0.40 ***	-	0.16 ***
Time	0.04	0.02	0.04	−0.03
Number of pets	0.00	0.01	0.00	0.01
Sex	−0.03	0.12	−0.02	−0.10
Variance of random components				
Random intercept	0.28 ***	0.14 ***	0.29 ***	0.23 ***
Residual variance	0.48 ***	0.40 ***	49 ***	0.39 ***
Direct effect, between-level	−0.05 CI 95% [−0.13, 0.03]		0.09+ CI 95% [0.00, 0.20]	
Direct effect, within-level	−0.03 CI 95% [−0.10, 0.03]		0.03 CI 95% [−0.04, 0.10]	
Indirect effect, between-level	−0.08 * CI 95% [−0.13, −0.04]		−0.03 * CI 95% [−0.07, −0.01]	
Indirect effect, within-level	0.02 * CI 95% [0.01, 0.04]		0.02 *** CI 95% [0.01, 0.03]	
AIC	4427.59		4469.50	
BIC	4449.79		4491.70	
-2LL	4419.59		4491.70	
R^2^	17.97		6.56	
Sample size	L_1_ = 1050; L_2_ = 105			

*Note*. Maximum likelihood estimation with robust standard errors (MLR) was used in the estimation. L_1_ = level 1, L_2_ = Level 2 analysis. *** *p* < 0.001, ** *p* < 0.01, * *p* < 0.05.

## Data Availability

Data will be made available upon reasonable request.
